# The Dilemma of Cure and Damage in Oligodendroglioma: Ways to Tip the Balance Away from the Damage

**DOI:** 10.3390/cancers10110431

**Published:** 2018-11-12

**Authors:** Ruurd Torensma

**Affiliations:** Department of Tumorimmunology, Radboud Institute for Molecular Life Sciences, RadboudUMC, Geert Grooteplein 28, 6525GA Nijmegen, The Netherlands; ruurd.torensma@radboudumc.nl; Tel.: +31-24-361-7600

**Keywords:** oligodendroglioma, single-cell RNA sequencing, pseudo-coloring MRI, Dendritic cell vaccination, fluorescence-guided surgery, mass spectrometry surgery, quality of life

## Abstract

Current treatments for oligodendrogliomas are powerful but have a negative impact on the rest of the body. The bone marrow is damaged by the chemotherapeutics, but other parts of the body are also affected. In this paper, the current treatment method and its collateral damage is described. Therefore, therapies are needed that are more effective against the tumor while having less negative effects on the patient’s quality of life. Some potential therapies include optimal removal of the tumor by fluorescent-guided surgery (FGS), intraoperative desorption electrospray ionization-mass spectrometry (DESI-MS), better monitoring of the effects of therapy by pseudo-coloring shades of gray of MRI pictures, and using recent data from RNA sequencing of single cells and immunotherapy. These are all open new ways of treating this tumor. The RNA sequencing of single tumor cells unravels specific tumor antigens present in the differentiation status of the cancer cell. Stem cell antigens were expressed in dividing cells, while hypoxia inducible factor-α (HIF-1α) is expressed in all tumor cells. Cancer stem cell antigens can be loaded on dendritic cells to induce cytotoxic T-cells directed to cancer stem cells. These recent discoveries suggest a better quality of life with the same overall survival.

## 1. Introduction

When a brain tumor is discovered, the first option is to remove the tumor by surgery. When possible, the patient is kept awake during surgery to enable interaction [1]. This prevents removal of healthy brain tissue that leads to unwanted damage. The tumor tissue that is obtained is of importance to establish the correct diagnosis and treatment. Since the tumor is interwoven with healthy brain tissue, the tumor is, in most cases, not completely removed. Although the MRI is used to monitor the tumor, other analysis methods demonstrate that MRI does not detect all parts of the tumor [2]. After surgery, even after photodynamic therapy or mass spectroscopy-based treatment, complete removal of all the tumor cells is yet not feasible and additional treatment is necessary. However, the more tumor tissue is removed, the longer the overall survival is [3,4].

**Figure 1:**
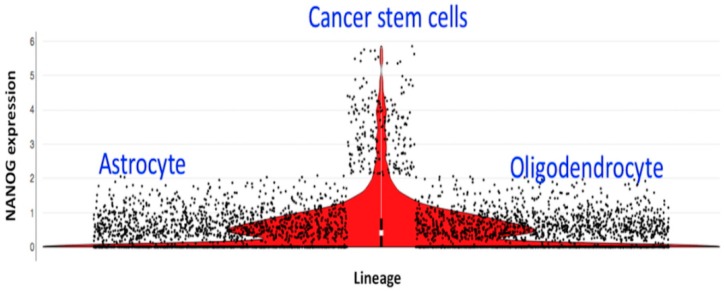
Violin plot of the differentiation hierarchy in oligodendrogliomas based on the single-cell RNA sequencing. Differentiation hierarchy based on differentiation scores (X) and stem cell scores (Y). Three distinct expression programs: oligodendrocyte (positive X, negative Y), astrocyte (negative X, negative Y), and stem cells (positive Y). The expression of NANOG in the different cells is indicated by a black dot. Principle component analysis of the RNA sequence of the single cells revealed two groups of cells with distinct lineage markers: astrocytes genes (137 genes for example APOE, ALDOC, SOX9 and oligodendrocyte genes (128 genes for example OLIG1, OLIG2, OMG. Cycling cells did not locate in both differentiated cell types and expressed stem cell genes. The algorithm allows to determine the expression of other RNA sequences in the basic violin plot. This analysis can be used online, at the Broad institute single cell portal. See for more information [11].

The current treatment of oligodendroglioma made a major step forward when several papers showed prolonged survival in patients receiving radiotherapy and chemotherapy (RTC) [5,6,7,8,9] as compared to either one of the treatments alone [10]. One explanation for the better overall survival of patients with oligodendroglioma after RTC is that cycling cells in oligodendroglioma are cancer stem cells (based upon the single-cell RNA sequencing [11], shown in Figure 1), and thus are susceptible to RTC [11,12].

**Figure 2:**
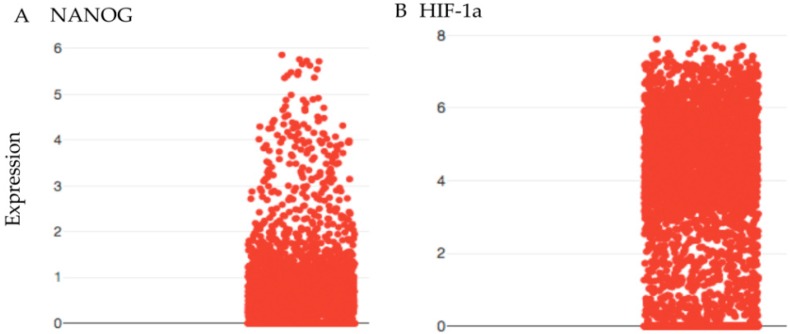
Box plot of single cell RNA sequencing of oligodendroglioma. Every dot is the expression of the chosen RNA for every single cell. (**A**) The RNA expression of NANOG is shown. Only a low number of cells show expression of NANOG (positive Y). (**B**) The RNA expression of hypoxia inducible factor-1α (HIF-1α; positive Y) is shown, which is expressed in most oligodendroglioma cells. RNA sequences were obtained from the online Broad Institute single-cell portal. At this moment, the portal is composed of 53 studies. For this paper the database with the RNA sequences from the Oligodendroglioma intra-tumor heterogeneity study were used.

Cancer stem cells feed the growth of the tumor and are the cause for recurrence of the tumor [12,13]. However, in oligodendroglioma, up to 4% of the undifferentiated (stem) cells are cycling cancer stem cells [14]. Therefore, 96% are cancer cells that are not cycling and are resistant to chemotherapy and RT [12]. In addition, 35% of undifferentiated cells are quiescent [14]. Oligodendroglioma is characterized by 1p/19q co-deletion and isocitrate dehydrogenase (IDH) mutation. The wild type enzyme converts isocitrate into α-ketoglutarate that enters the citric acid cycle for generating ATP. The mutated IDH converts isocitrate in 2-hydroxuglutarate that is unable to enter the citric acid cycle. The affected cell is unable to use the entire citric acid cycle, and this deprives the cell of getting enough ATP, leaving less energy to cycle at the normal rate. Evidence comes from single-cell RNA sequencing. We demonstrated that the hypoxia inducible factor (HIF-1α) is present in cancer stem cells as well in differentiated cancer cells, while cancer stem cells express NANOG, which is a stem cell marker (Figure 2).

This low cycling performance might be the reason that RTC is better than RT or chemotherapy alone, as there is less exposure of the therapeutics to the low number of cycling cells. Chemotherapy is abrogated from six cycles to three to four cycles due to the toxic effects even after a lower dose. Lowering the dose at the start of the course could result in a higher number of preset cycles and lead to more killing due to more encounters with cycling stem cells.

The benefit of RTC is substantial even after abrogating the prescript by six rounds and lowering the dose, with a median overall survival of more than 14 years on the shining side of the medal. The other side of the medal is less brilliant. The collateral damage is enormous, which is exemplified by the low number of patients that finish the prescribed six cycles of chemotherapy [5,6,15]. In the trials RTOG 9402 and EORTC 26951, the percentages of patients that received fewer cycles of therapy were 20% and 38%, respectively [16] even after lower doses. Significant hematological toxicities were observed in 56% and 46% of patients, respectively [16]. Recently, a paper reported that the median number for procarbazine cycles is three, and for CCNU (Lomustine) and vincristine this number is four [5]. Thus, the toxicity of the treatment is very high, leading to decreased dosing and prolonging cycle times, ranging from 42 to 56 days [5,9].

Hence, we set out to modify the treatment by using optimal surgical methods and use the power of the immune system that controls the cancer stem cell when it emerges.

## 2. Results and Discussion

The data were recovered from a patient that was treated with awake surgery followed with radiotherapy (52.2 Gy) and, after a recovery period, chemotherapy (PC) was started. Vincristine was deleted because it barely passed through the blood–brain barrier and, from the MRI with gadolinium, it appeared that the BBB was intact [17].

**Figure 3:**
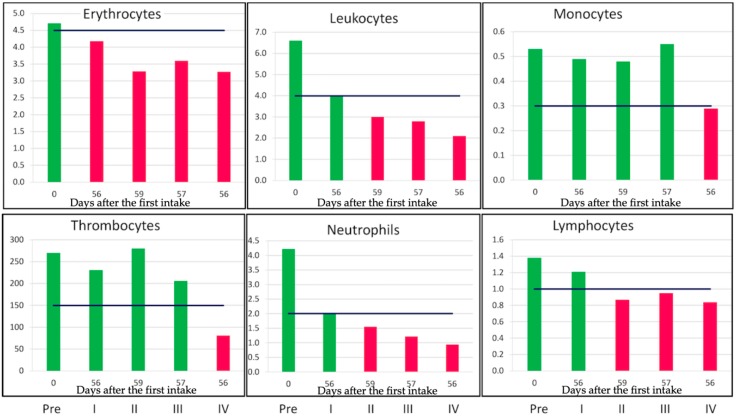
The level of blood cells at the different time point during chemotherapy. The levels are 10^12^/L for erythrocytes and the other cells are 10^9^/L. Pre: means the level before chemo is started. The four cycles are indicated by I, II, III, and IV. The cells are measured at the indicated days after the start of the cycle. The black line is the lower level of cells in a healthy person. Red is below this value and green is above this value. The roman numbers under the bars indicate the cycle number.

Even eight weeks after the start of the chemotherapeutics, the recovery of the bone marrow was not at the same level as the starting value (Figure 3).

The first two rounds of the standard dose (110 mg/m^2^ for CCNU and 60 mg/m^2^ for Procarbazine) were given. The last two rounds were given at 70% and 50% of the standard dose. However, by reducing the dose, the killing power is not reduced for hematopoietic cells. In Figure 3, the level of blood cell levels during four cycles is shown. The first bar represents the cell numbers before the start of the chemotherapeutics. Bar two and three are eight weeks after the start of the first and second cycle with the prescribed dose. In cycles three and four, the dose is reduced to 70% and 50% of the standard dose, respectively. At eight weeks, after the start of the fourth cycle, all blood cell types are below the minimal level of healthy persons (Figure 3). Even the recovery of thrombocytes takes more time. The data indicated that lower doses still have tremendous killing effects on hematopoietic cells. While a lower dose of chemotherapy still kills a proliferating blood cell, it is not clear if this also holds for the oligodendroglioma. At least for the bone marrow derived cells, one can conclude that reducing the dose by 30–50% does not result in more surviving bone marrow cells. At 280 days after the start of the fourth cycle, all cells were in the normal range, except for erythrocytes (4.17 × 10^12^/L), while hemoglobin was in the low normal range (8.8 mmol/L).

### 2.1. Cancer Stem Cells Are the Cycling Cells in Oligodendroglioma and the Targets of RTC

**Figure 4:**
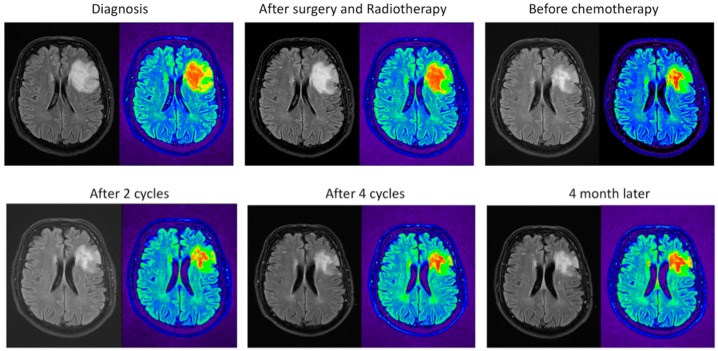
Pseudo-colored shades of gray of the MRI plots. Pseudo coloring was performed by the OsiriX Lite program by using the NIH palette. The slice that is depicted is from the highest tumor density. The highest intensity is colored red and are probably cancer stem cells, while the differentiated cells are colored green.

Single-cell RNA sequencing of oligodendrogliomas revealed a surprising feature. From single-cell RNA sequencing, it appeared that the cycling cells also expressed stem cell proteins (NANOG) while differentiated tumor cells did not (Figure 1) [11,18]. Apparently, transient amplifying cells are lacking. NANOG is a stem cell maintenance factor and is expressed in those cycling cells (Figure 1). Since cancer stem cells rarely divide and less than 4% will be in a cycle [11,14], it is expected that three cycles of RTC will hit more cancer stem cells but not all (Figure 4).

Additionally, the slow progression of the tumor indicates a low frequency of cycling.

**Figure 5:**
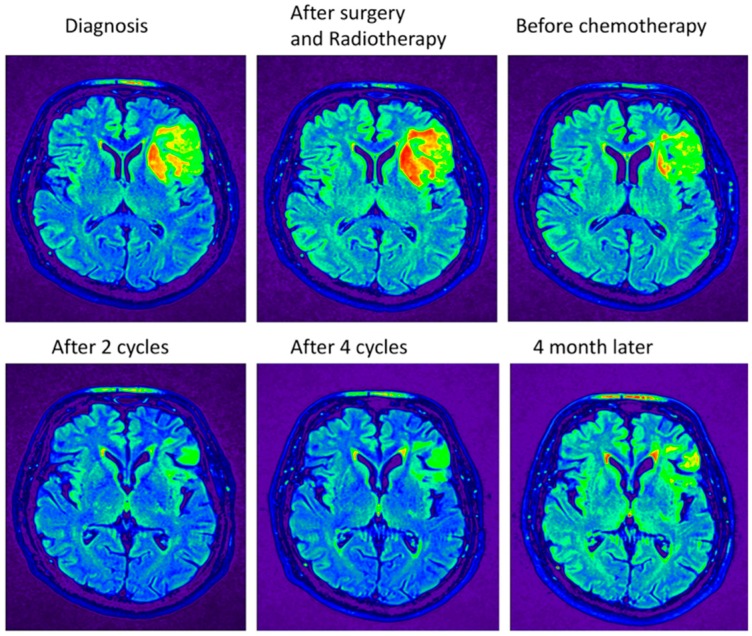
Pseudo-colored shades of gray of the MRI. The slice that is depicted is from the periphery of the tumor. The highest intensity is colored red and are probably cancer stem cells, while the differentiated cells are colored green.

**Figure 6:**
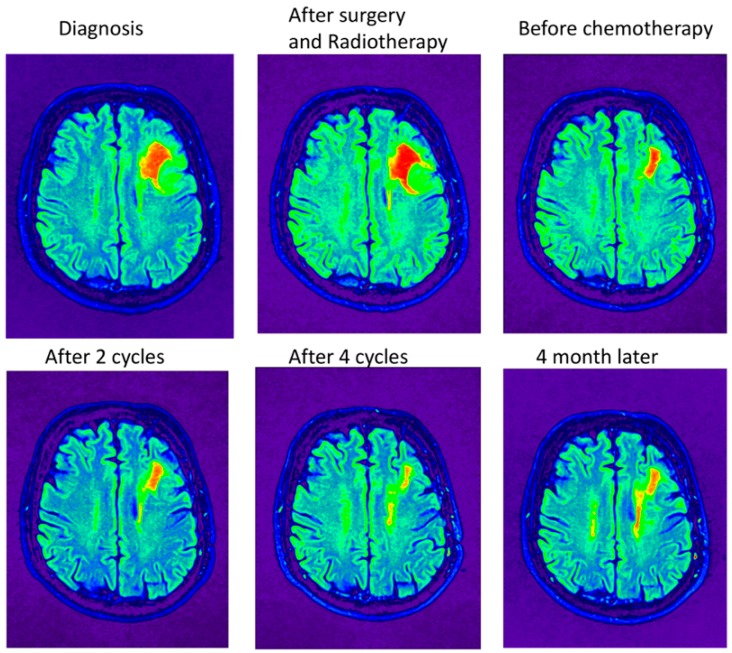
Pseudo-colored shades of gray of the MRI. The slice that is depicted is at the opposite site of the tumor. The highest intensity is colored red and are probably cancer stem cells, while the differentiated cells are colored green.

In general, all patients received the preset dose of radiotherapy. Radiotherapy alone gives a median overall survival of 7.3 to 9.7 years [6,7,16]. Although the tumor is treated only three or four times with chemotherapy, the overall survival is more than 14 years for the RTC-treated patients. The patient should benefit from receiving more cycles to kill the cancer stem cells. The MRIs taken before the chemotherapeutics were applied (including during the cycles and four months after the end of four cycles) still show tumor tissue (Figure 4). The tissue at the periphery shows the greatest reduction in size (Figure 5) while, deeper in the tumor, the reduction is less prominent (Figure 6).

Since cancer stem cells divide only once in a while, several cancer stem cells will escape killing when only three or four cycles are given. The hematopoietic stem cell divides only one time in one month [19]. It is assumed that the oligodendroglioma stem cell has an even slower cycling time due to the lack of sufficient ATP [14]. Almost all cancer cells including cancer stem cells express HIF-1α, which is a marker for hypoxia (Figure 2).

Hence, we are in a dilemma. The cancer stem cell that has a low pace in cycling needs more encounters with chemotherapeutic cycles to kill them all, but the patient resists such therapy because it decreases their quality of life [20]. Patients required a long recovery time for their blood cells to reach normal levels. Muscles take a long time to recover, which leads to a decline in physical well-being. However, incomplete recovery sometimes results in hearing loss, short-term memory loss, and loss of taste.

### 2.2. Diminishing the Damaging Effects by Lowering the Dose at the Start of the Chemotherapeutics

Lowering the dose is meant to prevent bone marrow toxicity. Seeing that the ruining effects on the patients’ fast dividing progenitors leads to stopping treatment, clinicians searched to other treatments. To decrease the toxic effects, temozolomide was recommended by clinicians [21]. However, for co-deleted oligodendrogliomas, Procarbazine, CCNU and Vincristine (PCV) demonstrated a better overall survival [22]. Others point to the same overall survival [10,23]. Since oligodendroglioma still has mostly an intact blood–brain barrier [17], vincristine could be deleted from the mix [16], especially because it causes neuropathy in a lot of treated patients [24].

Lowering the dosage is not enough for the blood cells to recover and to fulfill the six cycles. Apparently, lowering the dose still kills the bone marrow and the oligodendroglioma meets the remainder of the chemotherapeutics and leads to less killing of the tumor. MRIs were taken after several steps in the treatment. Pseudo-coloring shades of gray could help in the analysis. MRI pictures show the MRIs in gray and pseudo-colored (Figure 4). Figure 4 shows the MRIs in the midst of the tumor. There is hardly any tumor shrinkage during cycles with lower doses. The MRI after surgery and RT shows that it takes time to see the effects of the treatment, which is seen in the second and the third MRI in row one.

Apparently, the lower dose had a devastating effect on the bone marrow, while the effect on the oligodendroglioma was minimal.

Essentially, lowering the dose does not preserve the bone marrow and the oligodendroglioma where only a small shrinkage of the tumor is seen in the midst of the tumor. Actually, the penetration of the chemotherapeutic is less when the dose is lowered. To enable better killing of the tumor, the chemotherapeutic has to be delivered at the tumor and kept away from the bone marrow.

### 2.3. The MRI Monitors the Size of the Tumor

The oligodendroglioma tumor size is measured by monitoring and by sequential MRI imaging. However, the MRI images are not sharp and the intensity of the tumor is vague likely due to the fact that the healthy tissue and the tumor are interwoven.

As evident in Figure 4, the tremendous smaller size of the tumor due to the treatment is easily recognized when the intensity scales are pseudo-colored. However, it is clear that, after completion of the RT+PC using the standard schedule with a lowered dose, not all tumor cells are eradicated in the midst of the tumor. In the periphery of the tumor, more tumor cells are killed (Figure 5). The opposite site of the tumor shows a lower reduction, which indicates that there is less penetration with the lower dose (Figure 6). One has to conclude that bone marrow will take most of the dose and the remainder will enter the brain. If so, the brain will receive less of the chemotherapeutic, which leads to less damage to the cancer stem cell. Support for this explanation comes from in vivo practices. Unlike other chemotherapeutics, minimal hair loss is seen than with other chemotherapeutics. Apparently, even with the standard dose, the chemotherapeutics do not arrive in the skin area, which shows uneven distribution of the therapeutics. Therefore, one should aim to deliver first in the brain and less in the bone marrow.

### 2.4. Prospects for Optimal Tumor Removal by Surgery and Immunotherapy

As mentioned earlier, optimizing the sensitivity of the MRI could visualize the tumor and the removal of the tumor by surgery. However, the tumor is interwoven with normal brain tissue, which leaves in doubt if the surgeon can remove the whole tumor. Recently, several surgical procedures were described that use fluorescence of the tumor cell to remove more tumor cells [3,4,25,26,27,28,29]. They stated that the tumor is better when removed but, in spite of better surgery, tumors will unavoidably re-emerge [29].

Another method is to use mass spectrometry intra-operatively to remove as much of the tumor as possible [2,30,31,32,33]. However, the last method is still in its infancy but, for oligodendroglioma, a nice result is present because a tumor marker is available. Due to a mutation of isohydroxycitrate, dehydrogenase leads to 2-hydroxuglutarate, which is picked up by the mass spectrometer within minutes [30]. Recently, an update of this method was published. A MasSpec Pen was described adding to even better surgical removal of the tumor [34]. Awake surgery of the brain is yet another way to remove the tumor while sparing the healthy tissue [1,35]. However, awake surgery is not applicable to all patients due to anxiety and memories of the procedure [36].

### 2.5. Dendritic Cell (DC) Vaccination with Stem Cell Antigen

Cancer stem cells are the only cells in oligodendroglioma that lead to non-cycling tumor cells. The immune system is able to kill cancer stem cells in the brain when a cancer cell specific antigen is known. Such a procedure will save the bone marrow. The power of DC vaccination was recently described for breast cancer where the DC was loaded with mutated proteins [37]. For the neoantigen found in individual melanoma, a firm immune response was established [38]. In addition, glioblastoma DC therapy where the DC is loaded with cancer stem cell lysate shows remarkable results without affecting the bone marrow [39,40,41,42]. The first results of an autologous DC vaccine in a phase 3 clinical trial were recently reported [43]. Recently, we described a potential way to hit a cancer stem cell by directing the immune response to one of the stem cell antigens called NANOG [13]. The differentiated cells such as astrocytes and oligodendrocytes do not express cycling genes, which leaves only the cancer stem cell as a target for the immune system. In Figure 1, the stem cell and differentiation is depicted and the amount of NANOG that those cells express is also depicted. It is clear that the undifferentiated cell expresses the NANOG stem cell marker and several SOX genes [11,13]. Moreover, the immune system develops a memory response that is able to erase differentiated cells that re-emerge in the presence of cancer stem cells (plasticity).

## 3. Materials and Methods

### Patient Materials

Blood values and MRI pictures were collected and were in accordance with the guidelines and regulations of the Radboudumc.

The patient gave informed consent in concordance with the Declaration of Helsinki and the Netherlands Code of Conduct for Research.

## 4. Conclusions

Although the therapy for oligodendroglioma gives a long overall survival, it comes with a considerable burden for the patient. Lowering the dose does not prevent the bone marrow from being ruined, while the tumor is far less attacked. Antibody–drug conjugates (ADC) are a perfect way to deliver the drug at the cell to be killed. However, there are hardly any antigens to be found on cancer stem cells of oligodendroglioma that are cycling. Moreover, because of the low cycle time, the ADC has to be given several opportunities due to the low appearance of cycling stem cells. Recently, a method was described that targets antibodies to intracellular proteins [44] if the peptides are expressed in the MHC class I. The single-cell RNA sequencing revealed that the tumor is hypoxic due to the mutated IDH. All tumor cells express HIF-1α. This hypoxic state has a different hydrogen make-up than other normoxic cells and the hypoxic cells will light up in the MRI. A vaccine that targets the IDH mutant was able to generate a CD4 response against this neoantigen [45]. This enables us to clear the occupied space of differentiated non-cycling cells that, due to plasticity, can become cancer stem cells.

The recent additions of imaging during surgery are powerful but more research is needed. For example, a postoperative MRI suggested gross total tumor resection, while desorption electrospray ionization-mass spectroscopy (DESI-MS) detected tumor cells [2]. Better debulking of the tumor will diminish the plasticity of differentiated tumor cell to cancer stem cells [46,47]. Better removal of the tumor by these novel surgical methods followed by DC-NANOG immunotherapy could be a double-edged sword toward eradicating the cancer. The fueling cancer stem cell that drives tumor growth and the differentiated cancer cell that can revert to a cancer stem cell are controlled by an immunological memory of T-cells. Such blended therapies target the tumor without affecting healthy tissues. For patients, this makes a big difference and will lead to a better quality of life.
